# Cerebral Sinus Venous Thrombosis due to Asparaginase Therapy

**DOI:** 10.1155/2013/841057

**Published:** 2013-05-28

**Authors:** Youssef Alsaid, Shamshad Gulab, Mohammed Bayoumi, Saleh Baeesa

**Affiliations:** ^1^Department of Neurosciences, King Faisal Specialist Hospital and Research Centre, P.O. Box 40047, MBC J-76, Jeddah 21499, Saudi Arabia; ^2^Department of Hematological Oncology, King Faisal Specialist Hospital and Research Centre, P.O. Box 40047, MBC J-76, Jeddah 21499, Saudi Arabia; ^3^Division of Neurosurgery, Faculty of Medicine, King Abdulaziz University, Jeddah 21589, Saudi Arabia

## Abstract

We report a 9-year-old boy with acute lymphoblastic leukemia (ALL) in high-risk group who suffered from left sided focal seizures and ipsilateral hemiparesis during his induction with Asparaginase chemotherapy. Superior sagittal sinus thrombosis and right frontal hemorrhage were demonstrated on brain magnetic resonance imaging (MRI) scans . Anticoagulation was initiated with unfractionated heparin and switched to low molecular weight heparin after 3 weeks and continued for 6 months. At one-year followup, he had complete response to chemotherapy for ALL, with residual mild left hemiparesis, and his MRI scans revealed recanalized venous sinuses. The case highlights the importance of considering cerebral venous thrombosis as a complication of Asparaginase therapy.

## 1. Introduction

Cerebral venous sinus thrombosis (CVST) in children is rare. However, CVST is being increasingly recognized because of greater clinical awareness among clinicians, availability of sensitive neuroimaging techniques, and the survival of children with previously lethal diseases that confer a predisposition to sinovenous thrombosis [1]. One such predisposing condition is acute lymphoblastic leukemia (ALL) and its intensive induction chemotherapy.

The importance of chemotherapy in the pathogenesis of ALL-associated CVST is indicated by the observation that over 90% of cases occur during induction therapy; therefore, research has focused on chemotherapeutic agents administered and their influence on hemostasis [2]. 

Alterations in hemostasis have been well documented in children receiving Asparaginase as a single agent or in combination with prednisolone [2–5]. Cerebral venous sinuses thrombosis is a unique feature of Asparaginase-related thrombosis and is reported to occur in 1%–3% of patients [2]. 

Herein, we report a case of CVST in a 9-year-old boy undergoing induction chemotherapy for ALL. The correlation of CVST with hypercoagulable state, clinical-radiological features, and treatment are discussed.

## 2. Case Report

A 9-year-old boy presented to emergency department with headache and focal seizures of 1-day duration. He was newly diagnosed, on February 2012, with ALL and was just started his daily oral prednisolone, daunorubicin, weekly intravenous vincristine, and intrathecal chemotherapy. On day 3 of induction protocol, intramuscular polyethylene glycosylated- (PEG-) Asparaginase, the polyethylene glycol conjugate of *E. coli* L-Asparaginase (2500 IU/m^2^) was administered. 

His symptoms started on day 24 of induction protocol; he started complaining of severe headache and developed multiple episodes of left sided focal seizures. The seizures were of simple partial type and consisted of left sided clonic jerking. In addition, he was also complaining of right arm sensory disturbance. His birth and development periods were uneventful and there was no past history of convulsions. Parents were second-degree cousins and there was no history of epilepsy or stroke at young age in siblings or relatives. 

His examination revealed an alert and oriented child. Vital signs were normal. There were no signs of systemic illness. The neurological examination showed slurred speech. Cranial nerve examination showed left sided facial paralysis of upper motor neuron type. Motor examination revealed left sided hypotonia, power grade of 3/5, hyporeflexia, equivocal planters, and absent clonus. Motor findings were normal on the right side. There was no sensory deficit on examination.

**Figure 1:**
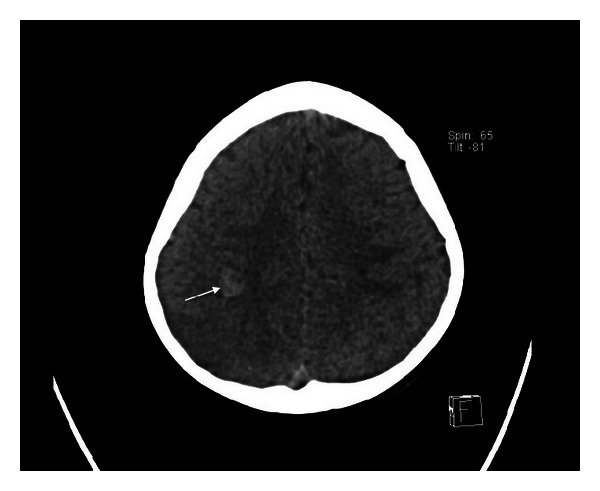
Plain CT scan demonstrated 10 mm × 15 mm right frontal intracerebral hemorrhage (arrow).

**Figure 2:**
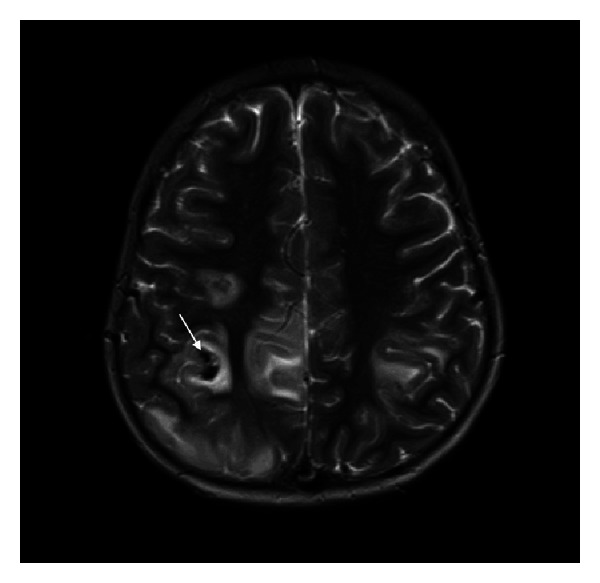
Axial T2-weighted MRI shows bilateral acute venous infarctions with hemorrhage (arrow) involving right frontal lobe.

**Figure 3:**
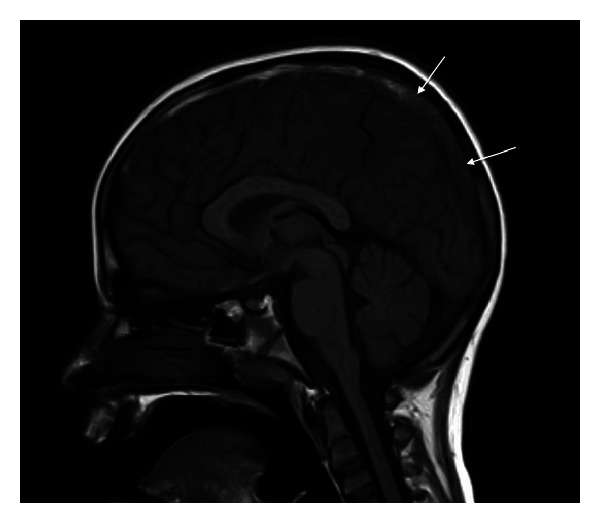
Sagittal T1-weighted MRI reveals low-attenuating thrombus (arrows) within superior sagittal sinus.

**Figure 4:**
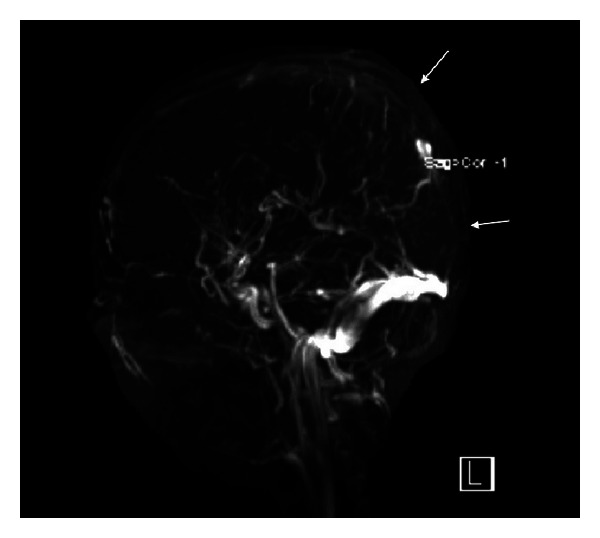
MR venogram showing absent flow signal in superior sagittal sinus (arrows).

The CT brain, performed within 24 hours of his presentation, showed subcortical hemorrhage (10 mm × 15 mm) in the right posterior frontal region (Figure 1). Brain magnetic resonance imaging (MRI), performed few hours after CT scan, showed bihemispheric multifocal hemorrhagic infarctions, more prominent on the right side (Figure 2), with associated significant vasogenic edema. There were hypointense signals within the venous structures with clear hyperintensity on T1 within the superior sagittal sinus (Figure 3). The postcontrast images showed a filling defect in superior sagittal sinus. The MR venogram (MRV) confirmed nonvisualization of superior sagittal sinus extending to the torcula (Figure 4). 

His coagulation profile in Table 1 showed deranged PT/APPT, elevated D-dimer, and low fibrinogen level. Factor VIII activity, antithrombin-III assay, proteins C and S, and homocysteine levels were normal. Anticardiolipin antibody profile, prothrombin gene mutation 20210, factor V Leiden, and methylenetetrahydrofolate reductase were negative. Fasting lipid profile was normal.

Initially he was substituted with fresh frozen plasma because of deranged coagulation profile and hypofibrinogenemia. Seizures were treated with levetiracetam 250 mg twice daily. Anticoagulation was initiated with intravenous unfractionated heparin (UFH) and after 3 weeks it was switched to low molecular weight heparin (LMWH). He developed severe persistent headache and mild papilledema due to intracranial hypertension for which an MRI brain was repeated to rule out hydrocephalus or hemorrhagic complications; it ruled out and responded well to oral acetazolamide (25 mg/kg/day) for 3 months. Heparin was discontinued after 6 months of this episode of CVST.

**Figure 5:**
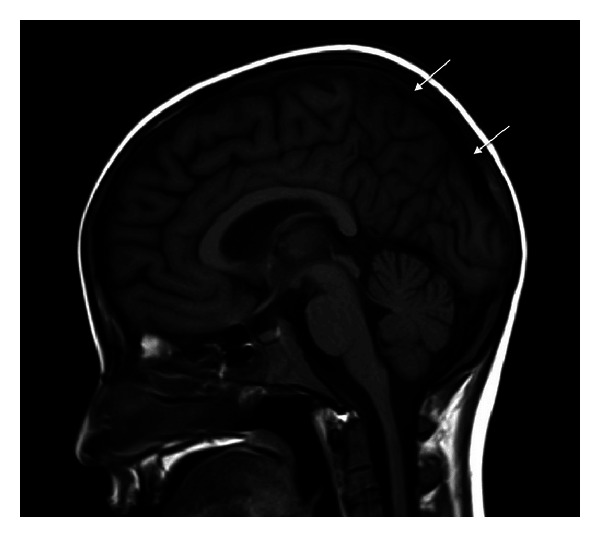
Sagittal T1-weighted MRI reveals normal flow signal indicating total resolution of superior sagittal sinus thrombosis (arrows).

**Figure 6:**
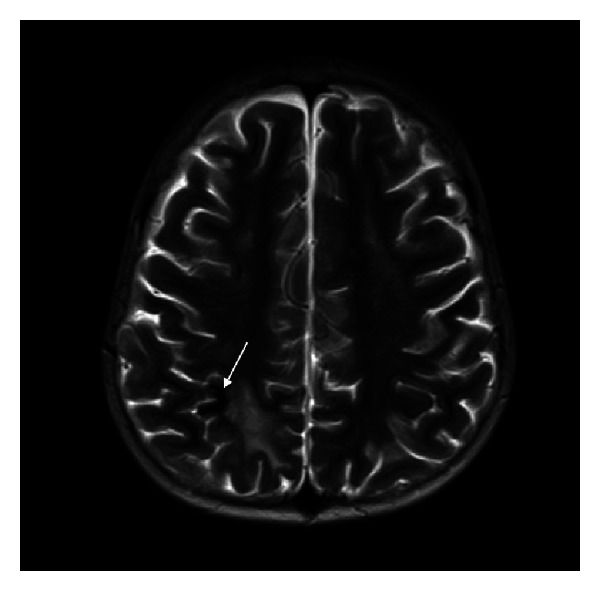
Axial T2-weighted MRI showing residual infarct with hemorrhage (arrow) in right frontal lobe with resolution of previous ischemic changes.

**Figure 7:**
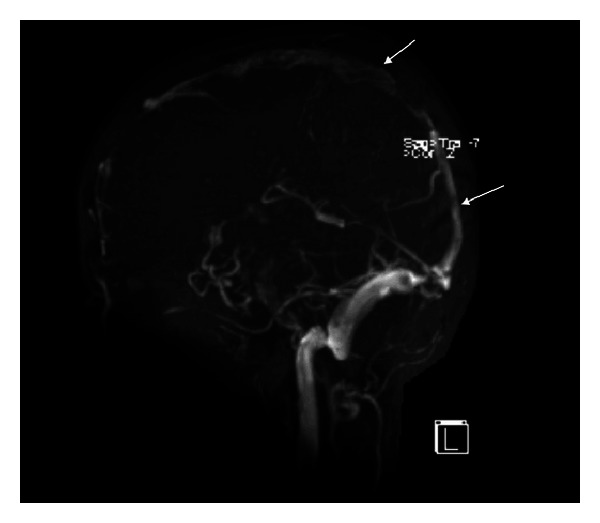
MR venogram showing complete resolution of the thrombus and reperfusion of the superior sagittal sinus (arrows).

On followup at 1 year, he was stable with mild persistent left sided grade 4 monoparesis and free from his primary disease. The follow-up MRI scans on February 2013 showed patent superior sagittal sinus with complete resolution of thrombosis, and small residual right frontal hemosiderin deposition from previous parenchymal hemorrhage (Figures 5, 6, and 7).

## 3. Discussion

Thrombosis in ALL is an invariably treatment related and often affects central nervous system [2]. The children at greatest risk are generally those receiving *E. coli* L-Asparaginase concomitant with prednisone [2–4]. Cerebral venous sinus thrombosis is a feature of Asparaginase-related thrombosis and has been reported in 1%–3% of treated patients [2]. Asparaginase-associated CVST usually occurs during induction; either during or within 2-3 weeks following therapy [2, 3, 6, 7].

Asparaginase is a bacterial-derived enzyme whose therapeutic effect in ALL has been well documented. It depletes Asparagine, thereby inhibiting protein synthesis in leukemic cells and the synthesis of many plasma proteins [5]. The latter effect reduces circulating levels of many hemostatic proteins including fibrinogen, plasminogen, and antithrombin-III. The deficiencies of these anticoagulants result in impaired inhibition of thrombin, which has been proposed as the main pathogenic mechanism for thrombosis [2, 3].

 Asparaginase preparation, length of exposure, and dose may play a role in thrombosis [2]. PEG-Asparaginase and L-Asparaginase both appear to have equivalent risk of thrombosis. One study demonstrated that the risk of cerebral thrombosis on PEG-Asparaginase was 3% [5]. The time interval between the dose and diagnosis of CVST in our case was about 3 weeks, which is consistent with previous reports of CVST with PEG-Asparaginase.

Besides Asparaginase, steroids are also used during induction therapy. Steroids reduce hepatic synthesis of coagulation proteins. The role of steroids alone in causing CVST is unclear, however, they may act in synergy with Asparaginase to increase the thrombosis by up to 6–8-folds [2–4].

Furthermore, children with underlying congenital prothrombotic disorders suffering from ALL have been shown to have higher risk of thrombosis on Asparaginase therapy [3, 8]. In our patient, thrombophilic screen was normal thus ruling out this risk factor.

The clinical manifestation of CVST, depending on site, size, and duration of thrombosis, can be seizures alone or in combination with headache, focal neurological deficits, and altered mental status [1, 9]. Bilateral symptoms, as seen in our patient, are rare but more characteristic. Diagnosis of CVST in the setting of ALL is typically based on clinical suspicion and neuroimaging confirmation. 

CT brain may show the direct signs of CVST in about one-third of the cases. The “dense triangle” sign is seen on noncontrast CT as a triangular hyperdensity in the posterior part of superior sagittal sinus, caused by the thrombus. The triangular defect (empty delta sign) from enhanced dura surrounding the thrombus can be demonstrated on contrast-enhanced CT. This finding may not appear for several days after symptom onset [1, 9–11], which explains the absence of it in our case. Indirect signs such as parenchymal infarcts with or without hemorrhages are more frequently depicted. 

CT venography and MRI with MRV are now the methods of choice for diagnosis of CVST. The principle early signs of CVST on noncontrast enhanced MRI are the combination of absence of a flow void with alteration of signal intensity in the sinus. In acute phase (3–5 days), thrombus appears isointense on T1 and hypointense on T2. This can be mistaken for normal flowing blood, and it may be necessary to use enhanced MRI or MRV to assist in the diagnosis. The thrombus is readily recognizable in subacute phase, when it is of high signal intensity on T1-weighted images. In our case, MRI brain was obtained a day after the symptom onset and yet the T1-weighted MR images showed hyperintensity, suggesting that symptoms appeared at least after 5 days of thrombosis. The indirect signs include parenchymal venous infarcts with or without hemorrhage, often bihemispheric and in nonarterial distribution. In an MRV, the direct signs of CSVT are high flow signal loss or fuzzy edges of a normally developed venous sinus or irregular lower blood flow signals [9–11]. 

The mainstay of management for CVST includes anticoagulation, and general supportive care. Currently, there are no evidence-based recommendations from randomized controlled trials on the use of anticoagulation in children. 

The American College of Chest Physicians (ACCP) Guidelines [12] for children with CVST and without significant intracerebral hemorrhage recommends anticoagulation with unfractionated heparin (UFH) or low molecular weight heparin (LMWH) followed by LMWH or warfarin for a minimum of 3 months. Additional 3 months are suggested if there is still cerebral sinovenous thrombosis or ongoing symptoms. For children with CVST and significant intracerebral hemorrhage, ACCP recommends either initial anticoagulation or radiologic monitoring alone at 5–7 days and anticoagulation if thrombus extension is noted at that time.

Some patients develop chronic headache due to elevated intracranial hypertension resulting from diminished CSF absorption, which may require long-term acetazolamide therapy, serial lumbar punctures, or lumboperitoneal shunting [9]; our patient responded to acetazolamide therapy alone, which was discontinued successfully after 3 months.

## 4. Conclusion

We emphasize that the occurrence of sudden onset of neurological symptoms and signs in the setting of ALL therapy, particularly with Asparginase, mandates ruling out CVST. Early diagnosis and prompt anticoagulation can minimize neurological morbidity. 

## Figures and Tables

**Table 1:** Laboratory parameters 3 weeks after PEG-Asparginase therapy.

Laboratory parameters	Reference ranges	Patient results
PT/INR	10.1–13.6 seconds/0.9–1.2	18.7/1.7 seconds
aPTT	24–36 seconds	39 seconds
D-Dimer	0–0.5 mg/L	1.67 mg/L
Fibrinogen	1.5–4 g/L	0.6 g/L
Factor VIII activity	0.5–2 IU/mL	>2 IU/mL
Protein C function	0.5–1.24 IU/mL	0.77 IU/mL
Protein S (total/free)	0.52–0.99/0.51–1.18 IU/mL	0.65/0.52 IU/mL
AT-III assay	70%–110%	93%
Anticardiolipin		
IgG	<10 GPL/mL	8 GPL/mL
IgA	<7 APL/mL	<2 APL/mL
IgM	<7 MPL/mL	<3 MPL/mL
Homocysteine	5–15 *µ*mol/L	3 *µ*mol/L
Fasting lipid profile		
Total cholesterol	0–5 mmol/L	3.9 mmol/L
Triglyceride	0–2.3 mmol/L	1.3 mmol/L
HDL	1–1.5 mmol/L	1.4 mmol/L
LDL	0–2.5 mmol/L	2.2 mmol/L

PT: prothrombin time, aPTT: activated partial thromboplastin time.
